# Nanoparticle-Decorated Ultrathin La_2_O_3_ Nanosheets as an Efficient Electrocatalysis for Oxygen Evolution Reactions

**DOI:** 10.1007/s40820-020-0387-5

**Published:** 2020-02-14

**Authors:** Guangyuan Yan, Yizhan Wang, Ziyi Zhang, Yutao Dong, Jingyu Wang, Corey Carlos, Pu Zhang, Zhiqiang Cao, Yanchao Mao, Xudong Wang

**Affiliations:** 1 Department of Material Sciences and Engineering, University of Wisconsin-Madison, Madison, WI 53706 USA; 2 Key Laboratory of Solidification Control and Digital Preparation Technology (Liaoning Province), School of Materials Science and Engineering, Dalian University of Technology, Dalian, 116024 People’s Republic of China; 3 MOE Key Laboratory of Materials Physics, School of Physics, Zhengzhou University, Zhengzhou, 450001 People’s Republic of China

**Keywords:** Oxygen evolution reaction, Multiphase hybrid, Two-dimensional nanomaterials, Rare-earth oxides, Ionic layer epitaxy

## Abstract

Electrochemical catalysts for oxygen evolution reaction are a critical component for many renewable energy applications. To improve their catalytic kinetics and mass activity are essential for sustainable industrial applications. Here, we report a rare-earth metal-based oxide electrocatalyst comprised of ultrathin amorphous La_2_O_3_ nanosheets hybridized with uniform La_2_O_3_ nanoparticles (La_2_O_3_@NP-NS). Significantly improved OER performance is observed from the nanosheets with a nanometer-scale thickness. The as-synthesized 2.27-nm La_2_O_3_@NP-NS exhibits excellent catalytic kinetics with an overpotential of 310 mV at 10 mA cm^−2^, a small Tafel slope of 43.1 mV dec^−1^, and electrochemical impedance of 38 Ω. More importantly, due to the ultrasmall thickness, its mass activity, and turnover frequency reach as high as 6666.7 A g^−1^ and 5.79 s^−1^, respectively, at an overpotential of 310 mV. Such a high mass activity is more than three orders of magnitude higher than benchmark OER electrocatalysts, such as IrO_2_ and RuO_2_. This work presents a sustainable approach toward the development of highly efficient electrocatalysts with largely reduced mass loading of precious elements.

**Electronic supplementary material:**

The online version of this article (10.1007/s40820-020-0387-5) contains supplementary material, which is available to authorized users.

## Highlights

- The 2.27-nm-thick hybridized quasi-2D structure of La_2_O_3_ crystalline nanoparticles embedded in La_2_O_3_ amorphous nanosheets (La_2_O_3_@NP-NS) exhibited a low overpotential of 310 mV at 10 mA cm^−2^.
- The mass activity of La_2_O_3_@NP-NS reached as high as 6666.7 A g^−1^ at overpotential of 310 mV. Such a high mass activity was more than three orders of magnitude higher than that of benchmark IrO_2_ (4.4 A g^−1^) and RuO_2_ (2.05 A g^−1^) and five orders of magnitude higher than that of commercial La_2_O_3_ (0.048 A g^−1^).

**Electronic supplementary material:**

The online version of this article (10.1007/s40820-020-0387-5) contains supplementary material, which is available to authorized users.

## Graphical abstract

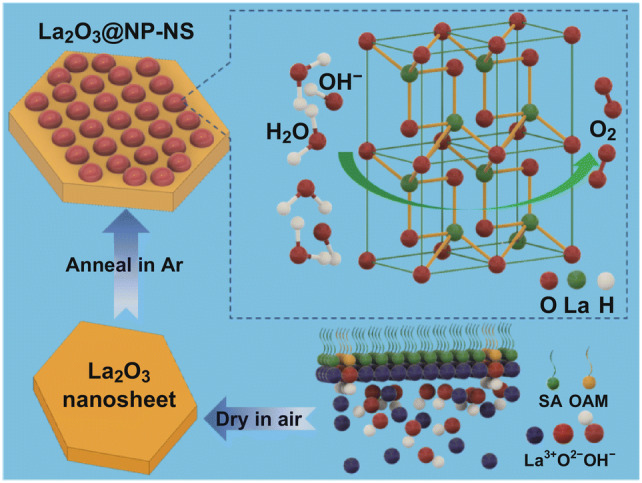

## Introduction

Today, oxygen evolution reaction (OER) is becoming an increasingly important process in many clean, renewable, and scalable electrochemical energy conversion and storage systems, such as fuel cells, electrochemical water splitting, solar fuel generation, and metal–air batteries [1–10]. To lead the exciting experimental developments of these energy systems to industrial applications, materials that catalyze OER with a high mass activity, a low overpotential, and a robust kinetic are highly desired [11–13]. Recent breakthroughs to lower the overpotential have revealed a large number of promising OER catalysts including carbon-based materials (e.g., graphene, CNT, and g-C_3_N_4_), and alternatives of transition metals (e.g., Mn, Co, Ni, and Fe) [13–25]. However, the low mass activity, high cost, and complicated fabrication procedure are still hindering scalable implementations of these materials in replacing the benchmark IrO_2_ and RuO_2_ that have high cost and limited supply [5, 13, 15, 26]. Therefore, development of high mass activity and high-efficient OER electrocatalysts based on earth-abundant elements is crucial and urgently needed to drive today’s advanced energy technologies forward [3, 13, 17].

Recently, rare-earth metal-based materials, especially rare-earth metal oxides, are found promising for a wide range of catalytic applications, including steam methane reforming, photocatalysis, water–gas shift reactions, thermochemical water splitting, and organocatalytic reactions [27]. Among them, La_2_O_3_ is showing a great potential as an alternative electrocatalytic candidate owing to its multiple oxidation states, excellent chemical stability, and low toxicity [27–29]. However, the electrochemical catalytic performance of commercial La_2_O_3_ powders is far from satisfaction mainly because of their low ratio of active catalytic sites and poor conductivity [5, 30]. Morphological alterations, particularly the two-dimensional (2D) geometry with just one or a few atomic layers, are a promising solution for improving the catalytic performance due to the abundant active sites, delocalized spin states, high electrical conductivity, and low mass loading [31, 32]. Furthermore, hybridization of 2D materials with nanoparticles (NPs) could offer an even greater boost to the electrochemical properties by combining the structural and electronic advantages of different morphologies [30, 33–35], such as lower the over potential of hydrogen evolution reaction (HER), and even raise the reversible capacity of lithium-ion batteries [36, 37]. Inspired by these previous advancements, reducing the dimension of La_2_O_3_ to a 2D structure and hybridizing with electrochemical active NPs may be a promising route leading La_2_O_3_ toward a high-performance electrochemical catalytic material in many energy conversion and storage systems. Based on this rationale, here, we report an ultrathin La_2_O_3_ nanosheets–NP hybrid structure (La_2_O_3_@NP-NS). The La_2_O_3_@NP-NS exhibited excellent OER performance with a low overpotential of 310 mV at 10 mA cm^−2^ and a very small Tafel slope of 43.1 mV dec^−1^ when the thickness of the La_2_O_3_ nanosheets was reduced to 2.27 nm. As a result of its uniform and ultrasmall thickness, they achieved a high mass activity, which was more than three orders of magnitude higher than benchmark IrO_2_ and RuO_2_, and five orders of magnitude higher than commercial La_2_O_3_ powder at the overpotential of 310 mV. This development presents an effective and scalable approach toward high-performance OER catalysts with a minimal use of precious rare-earth elements.

## Experimental

### Synthesis of 2.27-nm La_2_O_3_@NP-NS

Firstly, La_2_O_3_ nanosheets were synthesized by ionic layer epitaxy (ILE). In a typical process, 15 mL aqueous solution containing 0.2 mM La(NO_3_)_3_ and 2 mM hexamethylenetetramine (HMTA) was prepared in a glass vial with a 4.5 cm^2^ opening area. After standing the aqueous solution in air for 40 min, 10 µL chloroform solution of mixed surfactants containing (~ 0.9 vol %) stearic acid (SA) solution and (~ 0.1 vol %) oleylamine (OAM) solution was spread on the water surface. The SA solution had a concentration of 1.8 mol L^−1^. The OAM solution had a concentration of 1.8 mol L^−1^ and mixed with hydrochloric acid to a concentration of 0.01 mol L^−1^. This two-layer solution was exposed in atmosphere for 10 min to allow the chloroform to evaporate. Subsequently, the glass vial was screw-capped and placed in a 45 °C convection oven for 5 h for La_2_O_3_ nanosheets to grow. The La_2_O_3_ nanosheets were directly scooped onto a substrate and dried naturally in air. Secondly, the La_2_O_3_ nanosheets on the substrate were annealed in Ar atmosphere at 400 °C for 1 h, yielding 2.27-nm La_2_O_3_@NP-NS for further characterization and properties measurements. Si substrates were used for SEM and AFM characterization. Fifty-nanometer Au-coated Si substrates were used for XPS characterization. Holy carbon grids were used for TEM. The ILE-synthesized La_2_O_3_ samples were firstly transferred on holy carbon grids for TEM measurement, and these ILE-synthesized La_2_O_3_ samples on TEM grid were then annealed in Ar atmosphere for TEM analysis of La_2_O_3_@NP-NS samples. Fluorine-doped tin oxide (FTO) glass substrates were used for electrocatalytic OER measurement.

### Synthesis of Thicker La_2_O_3_ Nanosheets

La_2_O_3_@NP-NS (8.68 nm) and La_2_O_3_ (28.26 nm) nanosheets were synthesized through the same ILE and subsequent Ar annealing process as described above, where the reaction temperature during ILE process was increased to 60 and 80 °C for 5 h, respectively.

### La_2_O_3_ Nanoparticles–FTO Electrode

La_2_O_3_ NPs were synthesized under the same ILE process at a temperature of 80 °C for 5 h. The NPs were collected from the bulk solution by centrifuge. As-received La_2_O_3_ NPs (2 mg) was ultrasonically dispersed into an ethanol solution to form a catalytic ink. This ethanol solution contained 0.5 mL of ethanol and 50 μL of a Nafion^®^ solution (5 wt% in aliphatic alcohol from Sigma-Aldrich). La_2_O_3_ NPs–FTO electrode was prepared by depositing 3 μL catalytic ink onto surface of FTO, which corresponded to a loading of 0.02 mg of catalyst per cm^2^. The ink was dried at room temperature in air.

### IrO_2_–FTO, RuO_2_–FTO, and Commercial La_2_O_3_–FTO Electrode

Commercial IrO_2_, RuO_2_, or La_2_O_3_ powder (10 mg, from Sigma-Aldrich) was ultrasonically dispersed into an ethanol solution to form a catalytic ink. This ethanol solution contained 0.5 mL of ethanol and 50 μL of a Nafion^®^ solution (5 wt% in aliphatic alcohol from Sigma-Aldrich). IrO_2_–FTO or RuO_2_–FTO electrode was prepared by depositing 10 μL catalytic ink onto surface of FTO, which corresponded to a loading of 0.926 mg of catalyst per cm^2^. The ink was dried at room temperature in air.

### Material Characterizations

A Zeiss LEO 1530 field-emission scanning electron microscope was used to characterize morphologies of La_2_O_3_ nanosheets. The atomic force microscopy (AFM) tomography images were obtained using an XE-70 Park System. X-ray photoelectron spectroscopy (XPS) spectrum was obtained by a Thermo Scientific K-alpha XPS instrument with a 100 μm spot size, with the flood gun turned on during the measurements. The crystal structure was investigated by A FEI TF30 transmission electron microscopy (TEM) operated at 300 kV.

### Electrochemical Testing

The OER performance was done on an Autolab PGSTAT302N workstation with a standard three-electrode system, which was used to record the catalytic activity of samples in N_2_-saturated 1 M NaOH solution (PH = 13.6).The as-prepared La_2_O_3_@NP-NS on FTO substrate directly acted as the working electrode (mass loading: 0.0014 mg cm^−2^ for 2.27-nm La_2_O_3_@NP-NC, 0.0055 mg cm^−2^ for 8.68 nm La_2_O_3_@NP-NS, and 0.0186 mg cm^−2^ for 28.3 nm La_2_O_3_ nanosheets), the saturated Ag/AgCl was used as the reference electrode, and a platinum wire was used as the counter electrode. The OER polarization curves were recorded at a scan rate of 5 mV s^−1^ 1 M NaOH solution after purging with nitrogen for 30 min. All the potentials in this study are reported versus RHE. The electrochemical impedance spectroscopy (EIS) measurements were collected with frequencies ranging from 10 kHz to 0.1 Hz under an AC potential of 5 mV. For the stability test, the electrode was firstly activated in N_2_-saturated 1 M NaOH solution for 25 cycles and then recorded the first CV polarization curves at a sweep rate of 5 mV s^−1^. After continuous tests for 11 or 27 h, the final CV polarization curves were recorded at a sweep rate of 5 mV s^−1^ again. The overpotential (η) was calculated by the following relationship: *η* = *E* (vs. Ag/AgCl) + 0.21 *V* + 0.0592 × pH − 1.23 *V* − *iR*_u_, where *i* is the current, and *R*_u_ equals to the *R*_ct_ value resolved from Nyquist plots. TOF can be calculated as TOF = (*j* × *A*)/(4 × *F* × *n*), where *j* (mA cm^−2^) is the current density at a particular overpotential, *A* is the area of the working electrode, F is the Faraday constant (96,500 C mol^−1^), and n is the number of moles of the active materials. The mass activity can be calculated as: mass activity = *j/m*, where *m* is the mass loading of the working electrode (mg cm^−2^) and *j* is the measured current density (mA cm^−2^) at given potential. Electrochemical active surface area (ECSA) was calculated by the equation ECSA = *C*_dl_/*C*_s_, where *C*_s_ is the specific capacitance of the samples. Herein, *C*_s_ of 0.04 mF cm^−2^ was used according to the previously reported value of metal oxide/hydroxides in NaOH solution [38]. *C*_dl_ was calculated from the slope of the line in the plot of capacitive current density (*j*_dl_) versus scan rates *v* (V/s). *A* is surface area of electrode. *j*_dl_ = (*C*_dl_ × *ν*)/*A*, and cyclic voltammograms (CVs) were recorded in the double-layer regime (1 M NaOH, 0 to 0.1 V vs. Ag|AgCl) by varying scan rates ranging from 10 to 60 mV s^−1^.

## Results and Discussion

**Figure 1:**
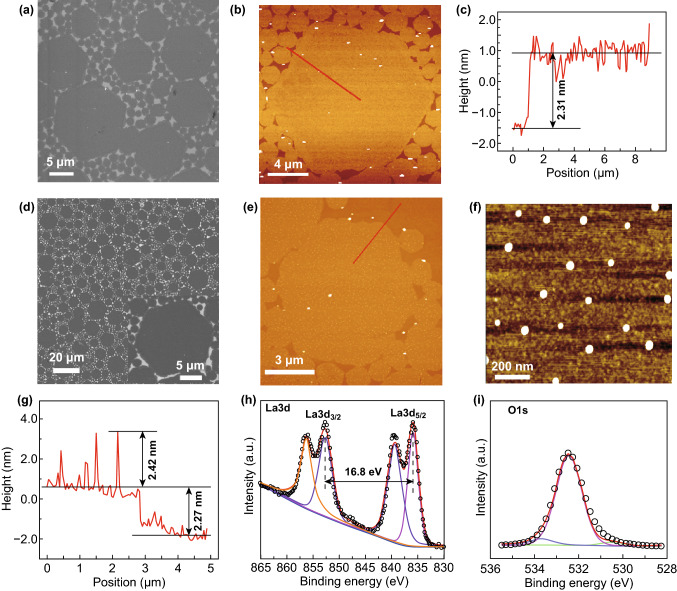
Morphology and elemental information of La_2_O_3_ nanosheets and La_2_O_3_@NP-NS. **a** SEM image of La_2_O_3_ nanosheets on a silicon substrate. **b** AFM topography scan of La_2_O_3_ nanosheets. **c** Height profiles derived from the red line in **b**. **d** SEM image of La_2_O_3_@NP-NS on a silicon substrate at low magnification. Inset is high-magnification SEM image of La_2_O_3_@NP-NS. **e** AFM topography scan of La_2_O_3_@NP-NS. **f** High-magnification AFM topography scan of La_2_O_3_@NP-NS. **g** Height profiles derived from the red line in **e**. **h** La 3*d* and **i** O 1*s* XPS spectrum of La_2_O_3_@NP-NS

The La_2_O_3_@NP-NS was synthesized by a solution-based ILE process at the water–air interface under a monolayer of mixed surfactants followed by Ar annealing as shown in Fig. S1. Firstly, the La_2_O_3_ nanosheets were synthesized by ILE process. This aqueous solution contained 0.2 mM La(NO_3_)_3_ and 2 mM HMTA as precursors and had a weak alkaline environment because HMTA decomposed into HCHO and ammonium hydroxide at 45 °C. Under weak alkaline conditions, La^3+^ could easily combine with OH^−^ to form Ce(OH)_3_, which would then be dehydrated to La_2_O_3_ after subsequent drying in the air. The as-synthesized La_2_O_3_ nanosheets exhibited a hexagonal shape with a bi-model size distribution (Fig. 1a). All the nanosheets appeared to be hexagonal, and their size exhibited a bi-model distribution. Larger nanosheets had a size of 15.7 ± 2.4 μm; while small ones were mostly around 1.8 ± 0.9 μm. This bi-model distribution was possibly due to the ILE mechanism where small nuclei or nanosheets tend to merge into big nanosheets driven by the reduction in surface energy [39]. Directly transferred La_2_O_3_ nanosheets covered almost the entire Si substrate surface, and no overlap was observed. AFM topography image (Fig. 1b) revealed that all the nanosheets had a very flat surface (surface roughness of 0.5 nm) with a uniform thickness of 2.31 nm (Fig. 1c). XPS analysis (Fig. S2a) showed that the spin–orbit splitting between La 3*d*_3/2_ and La 3*d*_5/2_ peaks was 16.8 eV, which was the typical characteristic of La_2_O_3_ phase [40–42]. TEM image (Fig. S3) revealed the amorphous structure of as-synthesized La_2_O_3_ nanosheets. In addition, the thickness of the La_2_O_3_ nanosheets could be tuned by the synthesis temperatures (Fig. S4). As the synthesis temperature rose from 45 to 60, and 90 °C, the nanosheet thickness increased from 2.31 to 8.73, and 28.30 nm, respectively. All the nanosheets still exhibited similar bi-model hexagonal geometry with excellent surface flatness.

The La_2_O_3_@NP-NS was obtained by annealing the as-synthesized La_2_O_3_ nanosheets in Ar at 400 °C for 1 h (Fig. S1). As shown by a low-magnification SEM image (Fig. 1d), the La_2_O_3_@NP-NS exhibited the same morphology and size distribution after the annealing. No cracks or dissociations could be observed. AFM image discovered that numerous fine NPs appeared on the nanosheet surface with a fairly uniform distribution (Fig. 1e). The surface density of the NPs was about 20 per μm^2^. Enlarged AFM image (Fig. 1f) showed that these NPs mostly had a spherical shape, anchored on the surface of nanosheets, and their sizes were in the range from 29 to 46 nm. The nanosheets still had a thickness of 2.27 nm (Fig. 1g), while the NPs were extruded above surface of nanosheet surface from 1 to 3 nm.

The elemental composition and chemical state of La_2_O_3_@NP-NS were then investigated by XPS. As shown in Fig. 1h, the La 3*d* spectrum was split into La 3*d*_3/2_ and La 3*d*_5/2_, located at 852.7 and 835.9 eV, respectively, with two satellite peaks at 856.3 and 839.4 eV, indicating the existence of La^3+^. Typical characteristic 16.8 eV between La 3*d*_3/2_ and La 3*d*_5/2_ peaks of La_2_O_3_ phase was still shown. In addition, the O 1*s* peaks at 532.5 and 530.5 eV could be assigned to the La–O bond in the La_2_O_3_ phase (Fig. 1i) [28, 43], further evidencing that the composition of La_2_O_3_@NP-NS was La_2_O_3_. By comparing the XPS results before and after Ar annealing, no obvious change was found for both La peaks and O peaks, suggesting both nanosheet and NPs had the same La_2_O_3_ chemistry. Thicker La_2_O_3_ nanosheets (8.73 and 28.30 nm) also showed the same morphology change after annealing (Fig. S5) with the same chemistry (Fig. S6). There was also a large quantity of NPs appeared uniformly on the surface of 8.68 nm nanosheets (Fig. S5c and inset). However, no NPs could be observed from the 28.26 nm nanosheets, possibly because the large thickness completely buried the NPs inside (Fig. S5d).

**Figure 2:**
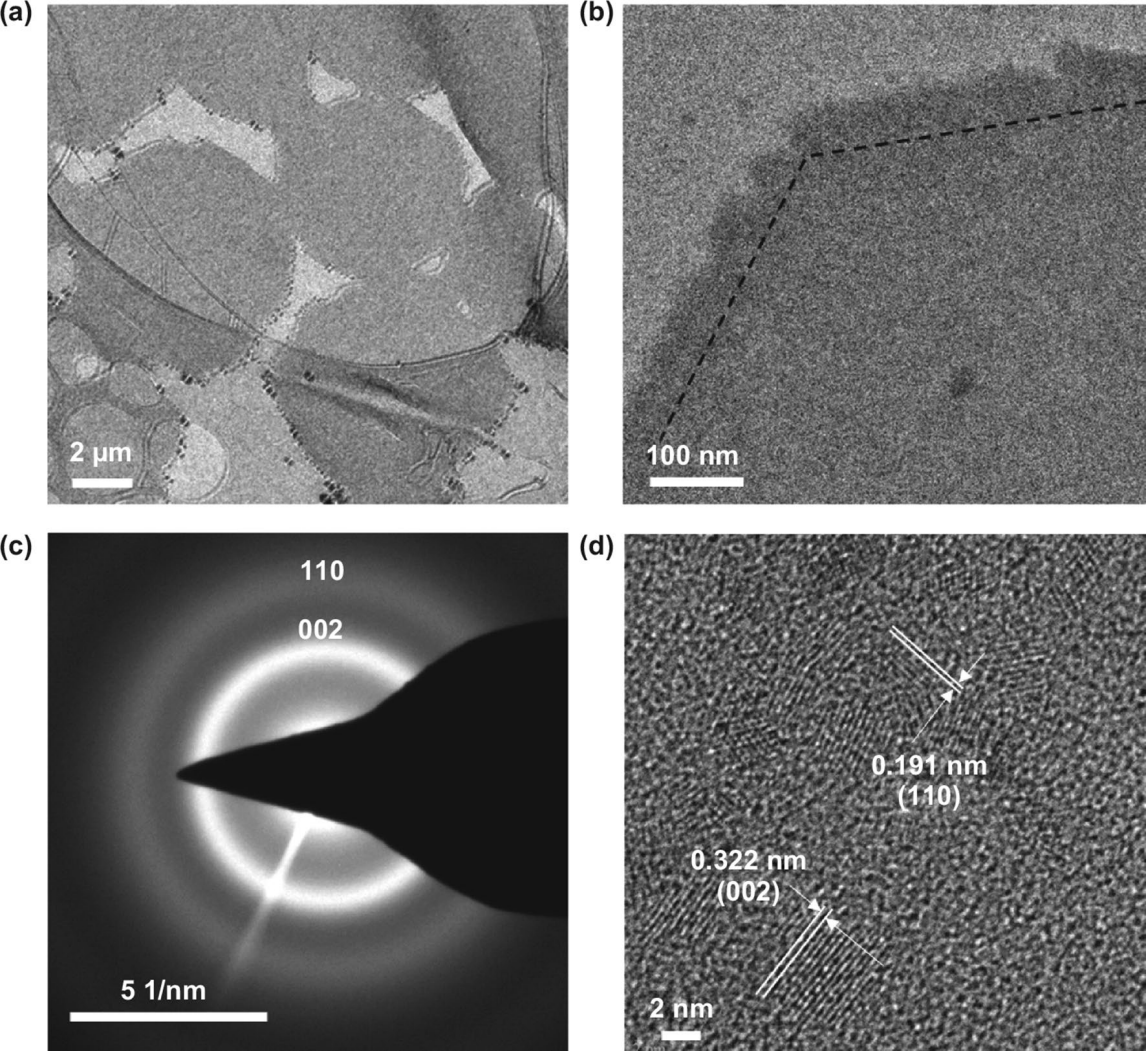
Structural characterization of 2.27-nm La_2_O_3_@NP-NS. **a** TEM image of hexagonal La_2_O_3_@NP-NS on a holy carbon TEM grid. **b** TEM image taken from a corner of a large La_2_O_3_@NP-NS. **c** SAED pattern of La_2_O_3_@NP-NS. **d** HRTEM image of La_2_O_3_@NP-NS

The crystal structure of La_2_O_3_ is hexagonal structure. There are two types of oxygen atoms in each hexagonal unit cell: One (denoted O4) is coordinated with four La atoms and the other (denoted O6) with six La atoms, while each La atom is coordinated with seven oxygen atoms: Four of them are O4, and the other three are O6 [44]. The crystal structure of La_2_O_3_@NP-NS was then studied by TEM. Low-magnification TEM image showed a uniform contrast of all hexagonal La_2_O_3_@NP-NS (Fig. 2a). A zoom-in image (Fig. 2b) at the corner area revealed that the angle between two adjacent edges of nanosheets was about 120° as marked by the dotted lines. The overall nanosheet showed a uniform contrast but no clearly observable lattices. The very front of all the edges was mostly wavy. Both features indicate amorphous or polycrystallinity structure of the nanosheet. Selected area electron diffraction (SAED) pattern collected from multiple nanosheets surfaces (Fig. 2c) showed wide and diffusive diffraction rings, suggesting that the La_2_O_3_@NP-NS indeed had a significant amount of an amorphous phase with small crystallites. The spread of interplanar spacing measured from the ring diameters was associated with the (002) and (110) planes of hexagonal-structured La_2_O_3_, confirming its polycrystalline structure [45]. HRTEM image (Fig. 2d) revealed the NP-NS hybrid structure. All the NPs had a crystalline phase with a size ranging from 6 to 32 nm, in good agreement with AFM observations. The interplanar spacing from the NPs was identified to be 0.324 and 0.192 nm, corresponding to the (002) and (110) planes of hexagonal La_2_O_3_ [45]. No crystal lattices could be observed from the nanosheet region, suggesting the nanosheet was still remained as an amorphous phase providing a uniform matrix supporting the crystalline NPs. These structure analyses indicated that during annealing in the Ar, the amorphous La_2_O_3_ nanosheets could firstly begin to crystallize and many tiny crystalline domains would thus form in the largely amorphous nanosheets; as the annealing time progressed, these tiny crystalline domains would then grow in both lateral and vertical size, gradually be extruded above surface of nanosheets and eventually grow into nanoparticles embedded in the nanosheets. Besides, Fig. S7 reveals the structure of crystallites distributed in the amorphous nanosheets region for 28.26 nm nanosheets, which confirmed that large thickness completely buried the NPs inside the 28.26 nm nanosheets after annealing.

**Figure 3:**
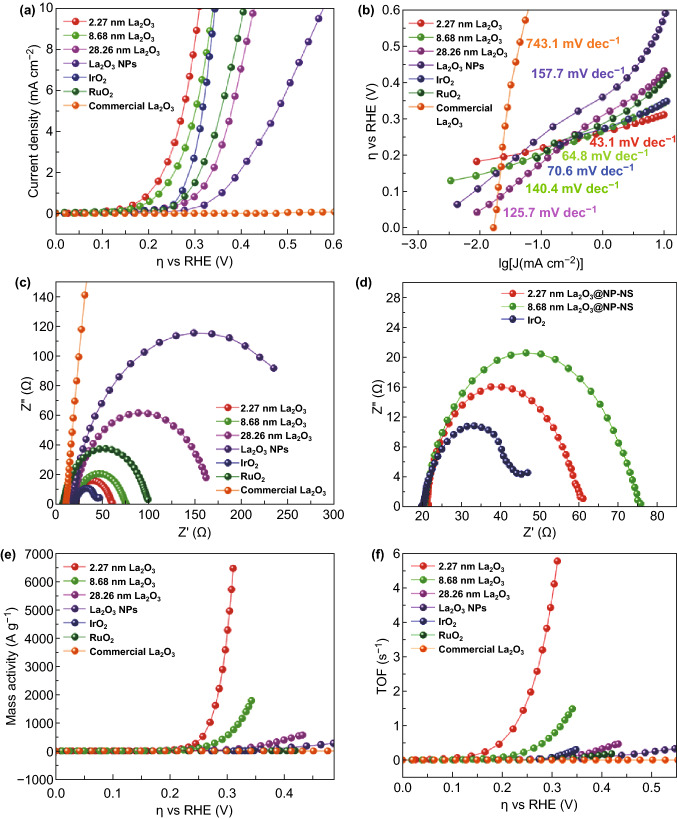
Electrocatalytic OER performance comparison of 2.27-nm La_2_O_3_@NP-NS (red), 8.68 nm La_2_O_3_@NP-NS (green), and 28.26 nm La_2_O_3_ nanosheets (magenta), ILE synthesized La_2_O_3_ NPs (violet), commercial IrO_2_ (blue), RuO_2_ (olive), and La_2_O_3_ powder (orange). **a** OER polarization curves measured in 1 M NaOH solution. **b** Tafel plots. **c** Nyquist plots measured in 1 M NaOH solution at a potential of 310 mV versus RHE. **d** Enlarged Nyquist plots. **e** Mass activity determined from current density as a function of *ŋ*. **f** TOF determined from *j* as a function of *η*. (Color figure online)

To investigate the electrocatalytic performance of La_2_O_3_@NP-NS for OER, the OER polarization curves of 2.27-nm La_2_O_3_@NP-NS on FTO substrate were probed in alkaline electrolyte (1 M NaOH). Through XPS analysis (Fig. S8), the La_2_O_3_@NP-NS grown on FTO substrate has the same La_2_O_3_ chemistry with that on Si substrate. As a comparison, the OER activities of thicker La_2_O_3_@NP-NS nanosheets, La_2_O_3_ NPs, commercial IrO_2_, RuO_2_, and La_2_O_3_ powders were also characterized under the same conditions. As shown in Fig. 3a, the 2.27-nm La_2_O_3_@NP-NS exhibited the smallest overpotential of 310 mV at a current density of 10 mA cm^−2^. Thicker nanosheets exhibited higher overpotential of 343 and 429 mV for 8.68 and 28.26 nm nanosheets, respectively. The very low overpotential from the 2.27-nm nanosheets outperformed the state-of-the-art noble metals oxides, e.g., IrO_2_ (343 mV) and RuO_2_ (408 mV) at the same current density. Besides, commercial La_2_O_3_ powders exhibited a very high overpotential of 1330 mV (Fig. S12a), confirming the significant morphological advantage of the 2D nanosheets.

Tafel plots from La_2_O_3_@NP-NS with different thicknesses and other comparison samples were then characterized to evaluate and compare their reaction rate constants and electrocatalytic kinetics. As illustrated in Fig. 3b, Tafel slope of the 2.27-nm La_2_O_3_@NP-NS was calculated to be only 43.1 mV dec^−1^, significantly lower than that of thicker nanosheets (Table 1). In addition, the low Tafel slope of the 2.27-nm La_2_O_3_@NP-NS was also superior to the benchmark IrO_2_, RuO_2_ (Table 1), and some recently reported novel OER catalysts, such as CeO_*x*_/CoO_*x*_ (66 mV dec^−1^) [46], NiO/CoN PINWs (44.5 mV dec^−1^) [47], Co_*x*_Ni_1−*x*_Fe_2_O_4_ (46.4 mV dec^−1^) [48], Ni_0.75_V_0.25_-LDH (50 mV dec^−1^) [49], and ZnCo_2_O_4_/N-CNT (70.6 mV dec^−1^) [50]. Such a low Tafel slope value from the 2.27-nm La_2_O_3_@NP-NS suggests that the reaction rate constant and charge transfer rate were greatly improved as the nanosheets thickness reduced to the ultrathin nanometer scale. These improved reaction rate constant and charge transfer rate could be attributed to 2D electron gas, which was possibly presented at the interface of NP-NS in the 2D La_2_O_3_ nanohybrids and contributed to their improved electrocatalytic properties by enhancing electron mobility and conductivity of nanohybrids [51].

**Table 1:** OER performance comparison between the samples synthesized in this work

Samples	Over potential (mV) @10 mA cm^−2^	Tafel slope (mV dec^−1^)	*R*_ct_ (Ω)	Mass activity (A g^−1^) @310 mV	TOF (s^−1^) @310 mV
2.27 nm La_2_O_3_@NP-NS	310	43.1	38	6666.7	5.79
8.68 nm La_2_O_3_@NP-NS	343	64.8	57	713	0.88
28.26 nm La_2_O_3_	429	125.7	143	51	0.055
ILE synthesized La_2_O_3_ NPs	580	157.7	272	17.4	0.012
Commercial IrO_2_ powder	343	70.4	25.4	4.4	0.0026
Commercial RuO_2_ powder	408	140.6	89.6	2.05	7.23 × 10^−4^
Commercial La_2_O_3_ powder	1330	743.1	47,598	0.048	4.87 × 10^−5^

EIS was further investigated to reveal the charge transfer resistance (*R*_ct_) of La_2_O_3_@NP-NS and to understand its role in the enhanced OER performance. As shown in Fig. 3c, Nyquist plot of the 2.27-nm La_2_O_3_@NP-NS had a much smaller semicircle compared to thicker nanosheets. From the Nyquist plots, *R*_ct_ of the 2.27-nm La_2_O_3_@NP-NS was found only about 38 Ω (Fig. 3d), which was lower than those of thicker nanosheets, La_2_O_3_ nanoparticles, and RuO_2_ powders, and only slightly larger than that of IrO_2_ powder (Table 1). The largely reduced *R*_ct_ was possibly due to the greatly shortened charge transfer distance between the catalysis surface and substrate as a result of the ultrasmall thickness [30].

One major advantage of the ultrathin nanosheet for electrochemical catalysis is its ultrahigh ratio of active catalytic sites. Figure 3e demonstrates the calculated mass activity plots. A remarkable mass activity of 6666.7 A g^−1^ was obtained from the 2.27-nm La_2_O_3_@NP-NS at overpotential 310 mV. It was one to more than two orders of magnitudes higher than other morphologies of La_2_O_3_ (Table 1) at the same overpotential. Such a large mass activity evidenced the unique merit of the ultrathin 2D geometry, which involved an extremely low mass and rendered considerably large exposed surface area allowing a very high ratio of catalytically active sites within the material. Besides, the NPs extruded above surface of nanosheet should also contribute to high ratio of active catalytic sites. Furthermore, this mass activity was three orders of magnitude higher than benchmark IrO_2_ (4.4 A g^−1^) and RuO_2_ (2.05 A g^−1^), and five orders of magnitude higher than commercial La_2_O_3_ at the same overpotential, suggesting that our 2.27-nm La_2_O_3_@NP-NS could be very competitive electrochemical catalysts for industrial applications. The electrochemical active surface area (ECSA) was also calculated based on cyclic voltammograms curves (Fig. S13) to estimate the exposure of active sites. As shown in Fig. S14, the values of ECSA for 2.27 (0.025) and 8.68 nm (0.021 cm^2^) La_2_O_3_@NP-NS were close and about twice as much as 28.26 nm La_2_O_3_ nanosheets, which could be because the NPs extruded above surface of nanosheet also contributed to active catalytic sites. Moreover, the ECSA value of commercial La_2_O_3_ powder (0.31 cm^2^) was much bigger than that of 2.27-nm La_2_O_3_@NP-NS, but it exhibited poor OER performance, which could attribute to good conductivity caused by ultrathin thickness, but poor intrinsic OER catalytic activity of commercial La_2_O_3_ powder [32].

The turnover frequency (TOF) was another critical OER parameter that evaluates the number of O_2_ molecules formed per active metal site per second. As shown in Fig. 3f, the TOF of 2.27-nm La_2_O_3_@NP-NS reached up to 5.79 s^−1^ at the overpotential of 310 mV, which was about one to five orders of magnitudes higher than those comparison samples (Table 1). The extremely high TOF could be attributed to the largely facilitated electron transfer from the ultrathin nanosheet to the FTO substrate as well as the very large mass activity. The significant enhancement in both mass activity and TOF confirmed the ultrathin La_2_O_3_@NP-NS could provide a highly efficient OER performance with a minimal use of rare-earth elements.

To further figure out the origin of the active sites for La_2_O_3_@NP-NS, electrocatalytic OER performances of 2.31 nm La_2_O_3_ nanosheets before Ar annealing were also investigated and compared to that of 2.27-nm La_2_O_3_@NP-NS, as shown in Fig. S15. It was found that the OER performance of 2.31 nm La_2_O_3_ nanosheets was greatly enhanced after Ar annealing at 400 °C for one hour. The overpotential was shifted toward a lower overpotential by 59 mV after annealing, with corresponding Tafel slope reducing from 95.4 to 43.1 mV dec^−1^. Moreover, mass activity increased greatly from 3238 to 6666.7 A g^−1^. All these results confirmed that the catalytic active sites of La_2_O_3_@NP-NS were attributed to precipitated La_2_O_3_ nanoparticles after Ar annealing, as well as ultrathin nanosheet region.

To better understand the OER performance of the ultrathin La_2_O_3_@NP-NS hybrid structure, the OER characteristic data (e.g., overpotential, mass loading, mass activity, and TOF) from other representative state-of-the-art OER catalysts were collected and compared (Table S1). While the overpotential from this work was generally superior or at least comparable to those Ni-, Co-, Mn-, or Fe-based OER catalysts, the biggest advantage of our nanosheets was obviously the extremely large mass activity. As a result of the ultrasmall thickness and uniform coverage over a large area, the mass loading of our La_2_O_3_@NP-NS was only 0.0014 mg cm^−2^, which brought the mass activity to more than three orders of magnitude higher than typical OER catalysts such as CoFe–LDHs [52], Co_3_O_4_ [53], and Ni@NC [54].

**Figure 4:**
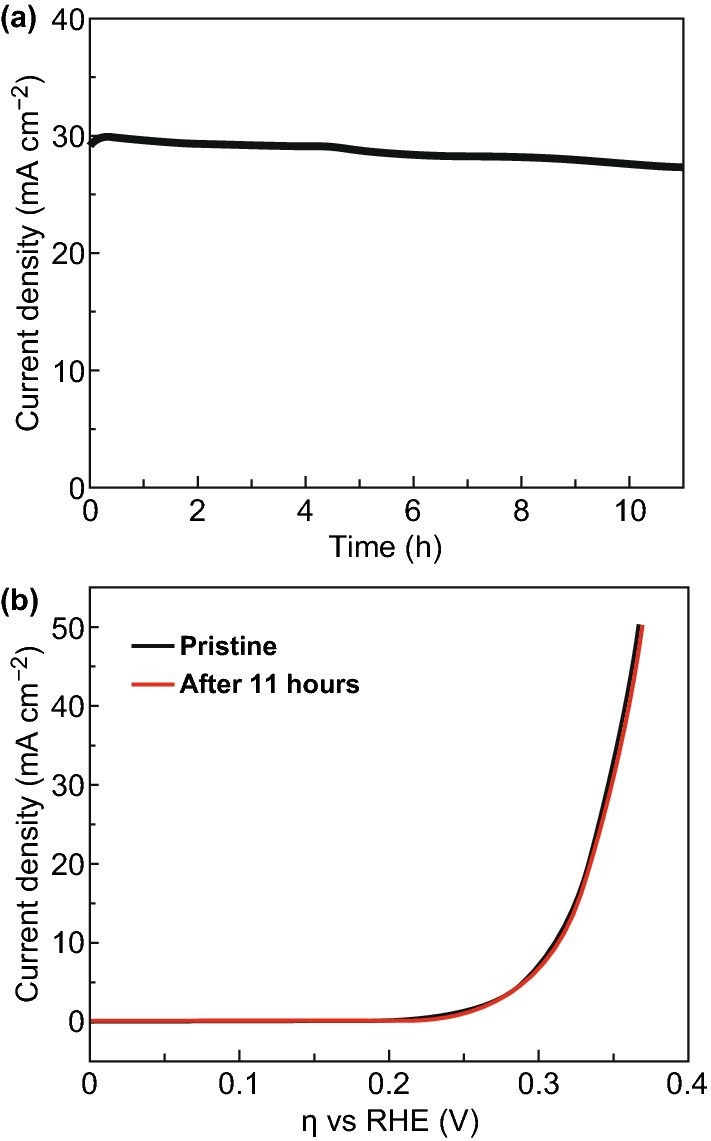
Electrochemical stability tests. **a** Current density measured at *η* = 345 mV (vs. RHE) as a function of time. **b** OER polarization curves of 2.27-nm La_2_O_3_@NP-NS before and after 11-h OER

For nanostructured catalysts, the stability is vital due to the largely increased atom mobility on nanoscale surfaces. Impressively, the ultrathin La_2_O_3_@NP-NS exhibited superb stability with 90% retention of the initial current density after 11 h of continuous OER operation in 1 M NaOH at *η* = 345 mV (Fig. 4a). SEM image showed that before (Fig. S16a) and after (Fig. S16b) the 11-hour continuous OER test, the coverage of nanosheets was nearly the same and no obvious damage could be observed; XPS analysis (Fig. S17a) revealed that the La_2_O_3_@NP-NS almost retained their chemistry. In addition, the OER polarization curve showed only a slight decay after the OER test (Fig. 4b), which confirmed appropriate function of the La_2_O_3_@NP-NS coating during the long-term operation. The high stability of the ultrathin La_2_O_3_@NP-NS nanosheets might be attributed to the amorphous–crystallites hybrid structure. The amorphous La_2_O_3_ matrix could stabilize the small La_2_O_3_ NPs from aggregation by limiting the atomic diffusion, and on the other hand, the anchored La_2_O_3_ NPs could act as spacers to prevent the 2D La_2_O_3_ nanosheets from restacking during electrochemical cycling [37, 51]. Besides, the stability test of 2.27-nm La_2_O_3_@NP-NS with longer time was also investigated. As shown in Fig. S18a, the stability test survived for 27 h because there was a sharp decrease in the current density at the time range from 25 to 27 h. SEM images (Fig. S18c, d) revealed that the majority of La_2_O_3_@NP-NS fell off and only part of samples was adsorbed on the FTO substrate after 27 h stability test, which resulted in the decrease in current density during stability test and confirmed that OER performance truly came from the ultrathin La_2_O_3_@NP-NS. Long-hour immersion in alkaline solution may weaken the adsorption of La_2_O_3_@NP-NS on FTO substrate, which could cause the La_2_O_3_@NP-NS to fall off from the substrate during stability test.

## Conclusions

In conclusion, La_2_O_3_@NP-NS was synthesized by a facile solution-based ILE approach in a large area. Outstanding electrocatalytic OER performance was observed when the nanosheet thickness was reduced to 2.27 nm. The 2.27-nm La_2_O_3_@NP-NS exhibited a low overpotential of 310 mV at 10 mA cm^−2^, a small Tafel slope of 43.1 mVdec^−1^, and charge transfer resistance of 38 Ω. A high mass activity of 6666.7 A g^−1^ and turnover frequency of 5.79 s^−1^ were also obtained from the 2.27-nm La_2_O_3_@NP-NS at an overpotential of 310 mV. This very large mass activity was more than three orders of magnitude higher than benchmark IrO_2_ (4.4 A g^−1^) and RuO_2_ (2.05 A g^−1^), and five orders of magnitude higher than commercial La_2_O_3_ (0.048 A g^−1^) at the same overpotential. The nanosheets also exhibited an impressive stability for over 11-h continuous operation with only 10% decay. The excellent electrocatalytic OER activity could be attributed to the ultrathin thickness that enables overall short out-of-plane charge diffusion length and facilitated electron transfer from the nanosheet surface to the substrate. This development opens up a promising strategy for the development of highly efficient electrocatalysts with largely reduced loading of catalytic materials. Creating the hybrid ultrathin 2D morphologies could potentially become an ultimate solution to preserve precious elements in advanced and sustainable electrocatalyst design in many energy and environmental systems.

## Electronic supplementary material

Below is the link to the electronic supplementary material.

Supplementary material 1 (PDF 1875 kb)
