# Supplementary material for: Nanoparticle-Decorated Ultrathin La2O3 Nanosheets as an Efficient Electrocatalysis for Oxygen Evolution Reactions
Source: Nanomicro Lett. 2020 Feb 14;12:49. doi: 10.1007/s40820-020-0387-5 (PMC7770806; doi:10.1007/s40820-020-0387-5)
Supplement: Supplementary file 1 — Supplementary material 1 (PDF 1875 kb) [file 40820_2020_387_MOESM1_ESM.pdf]

Supporting Information for

Nanoparticle-Decorated Ultrathin La_2O_3 Nanosheets as An Efficient Electrocatalysis for Oxygen Evolution Reactions

Guangyuan Yan[1, 2], Yizhan Wang[1], Ziyi Zhang[1], Yutao Dong[1], Jingyu Wang[1], Corey Carlos[1], Pu Zhang[3], Zhiqiang Cao[2, *], Yanchao Mao[3, *], Xudong Wang[1, *]

[1]Department of Material Sciences and Engineering, University of Wisconsin-Madison, Madison, WI 53706, USA

[2]Key Laboratory of Solidification Control and Digital Preparation Technology (Liaoning Province), School of Materials Science and Engineering, Dalian University of Technology, Dalian 116024, People's Republic of China

[3]MOE Key Laboratory of Materials Physics, School of Physics, Zhengzhou University, Zhengzhou 450001, People's Republic of China

*Corresponding authors. E-mail: xudong.wang@wisc.edu (Xudong Wang); caozq@dlut.edu.cn (Zhiqiang Cao); ymao@zzu.edu.cn (Yanchao Mao)

Supplementary Figures

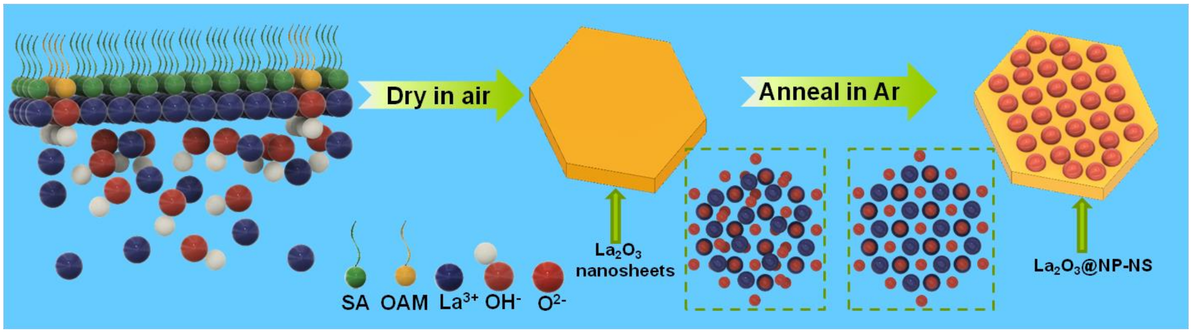

Fig. S1 Schematic of synthesis process of La_2O_3@NP-NS

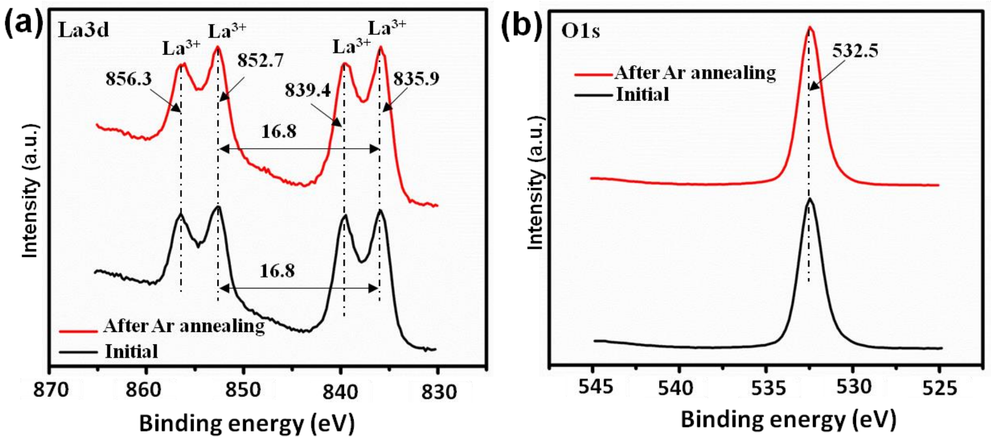

Fig. S2 a La 3d and **b** O 1s XPS spectrum of 2.27 nm La_2O_3@NP-NS before and after Ar annealing

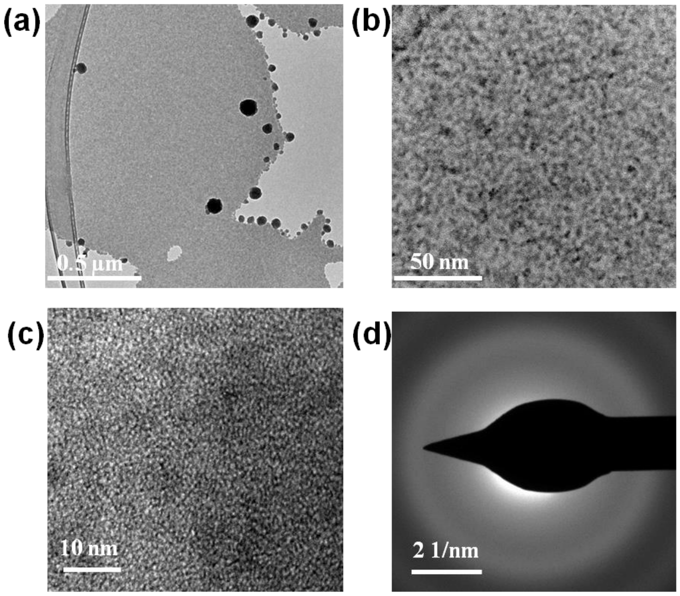

Fig. S3 Structural characterization of La_2O_3 nanosheets before annealing. **a** TEM image of hexagonal La_2O_3 nanosheets. **b-c** HRTEM images of La_2O_3 nanosheets. **d** Corresponding SAED pattern of La_2O_3 nanosheet

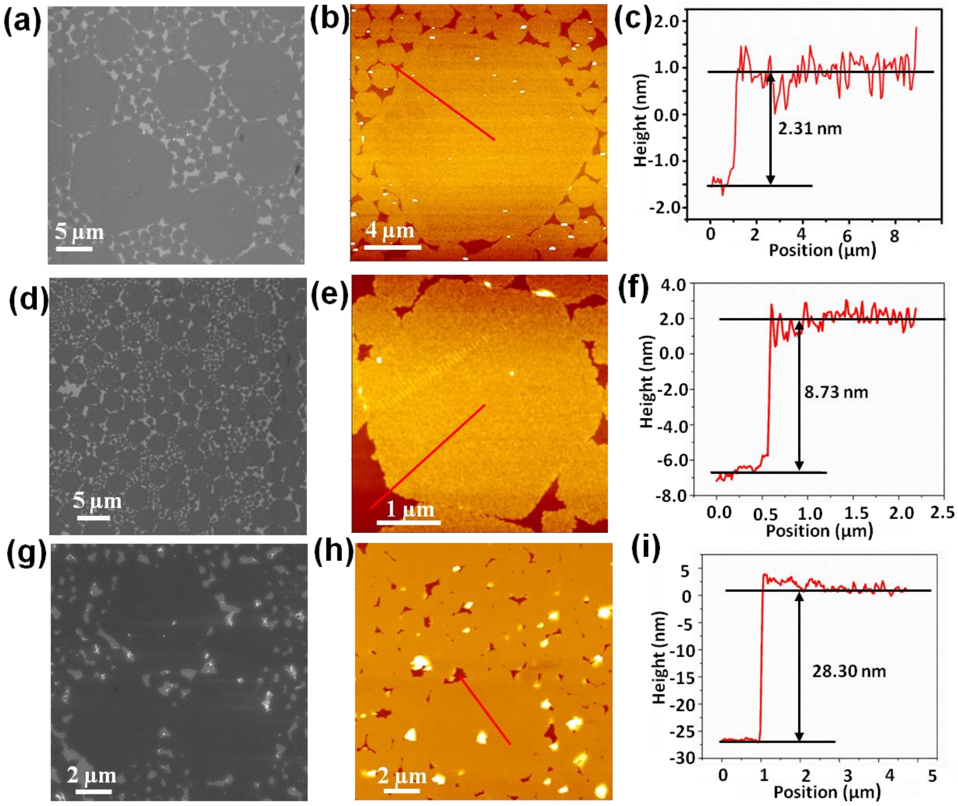

Fig. S4 La_2O_3 nanosheets grown for 5 h at **a-c** 45 °C, **d-f** 60 °C, **g-i** before annealing: **a**, **d**, **g** SEM images, **b**, **e**, **h** AFM topography images, and **c**, **f**, **i** height profiles along the red dashed lines in corresponding AFM topography images

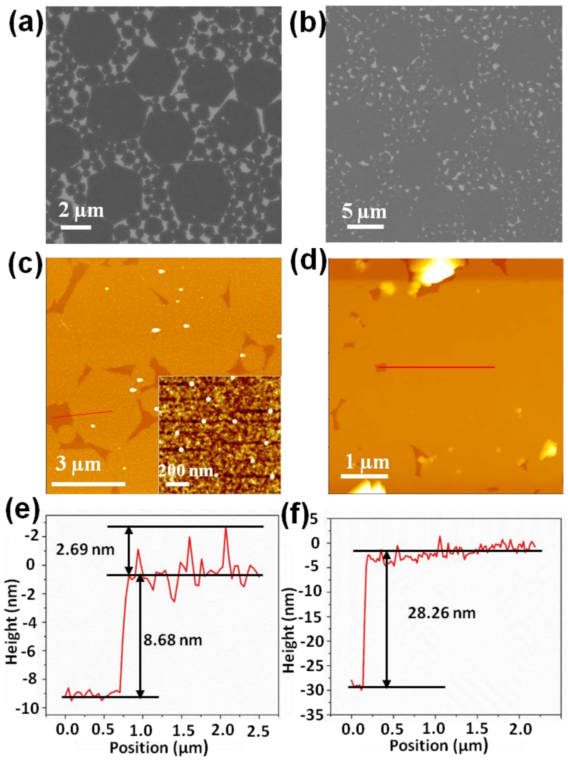

Fig. S5 Morphology and thickness of 8.68-nm La_2O_3@NP-NS and 28.26-nm La_2O_3 nanosheets after Ar annealing. **a**–**b** SEM images, **c**–**d** AFM topography images, inset in **c** is high-magnification AFM topography scan of nanosheets, and **e**–**f** height profiles along the red dashed lines in corresponding AFM topography images of nanosheets grown for 5 hours at **a**, **c**, **e** 60 °C, **b**, **d**, **f** 80 °C

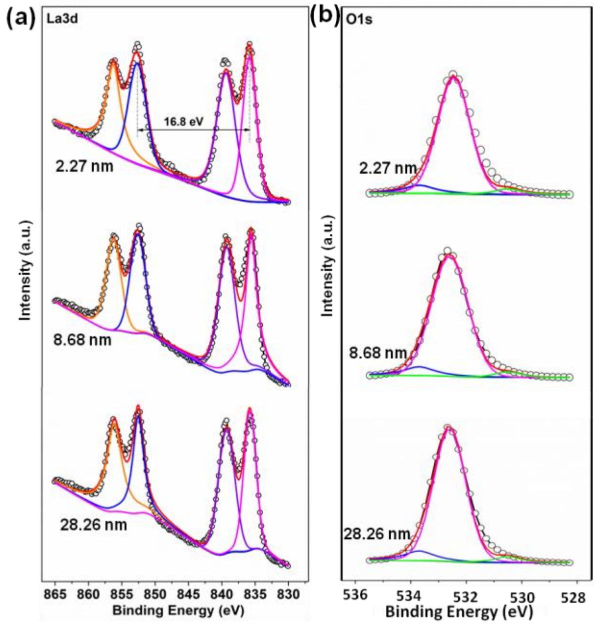

Fig. S6 Fitted XPS spectrum of characteristic X-ray peak of La 3d **a** and O1s **b** from 2.27-nm La_2O_3@NP-NS, 8.68-nm La_2O_3@NP-NS, and 28.26-nm thick La_2O_3 nanosheets

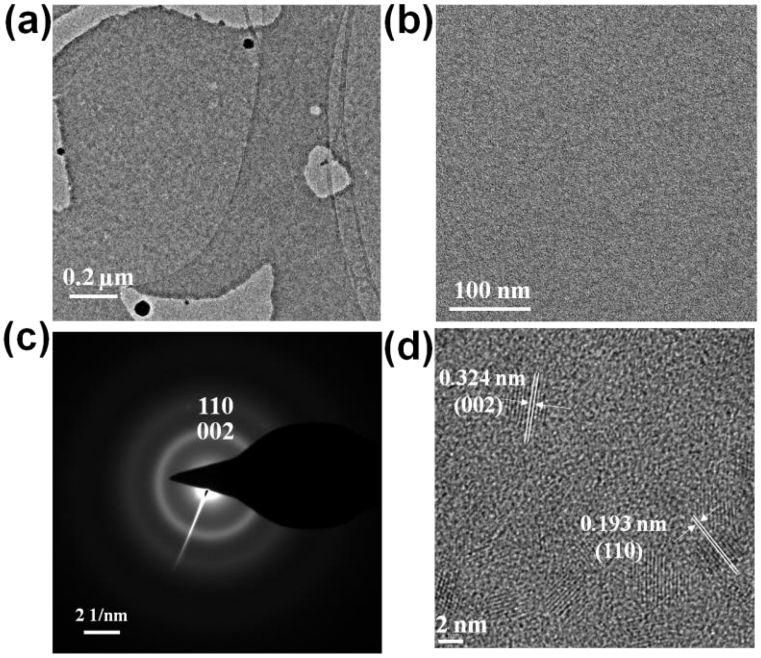

Fig. S7 Structural characterization of 28.26-nm La_2O_3. **a** Low-magnification TEM image of hexagonal La_2O_3 nanosheet on a holey carbon TEM grid. **b** High-magnification TEM image. **c** SAED pattern. **d** HRTEM image

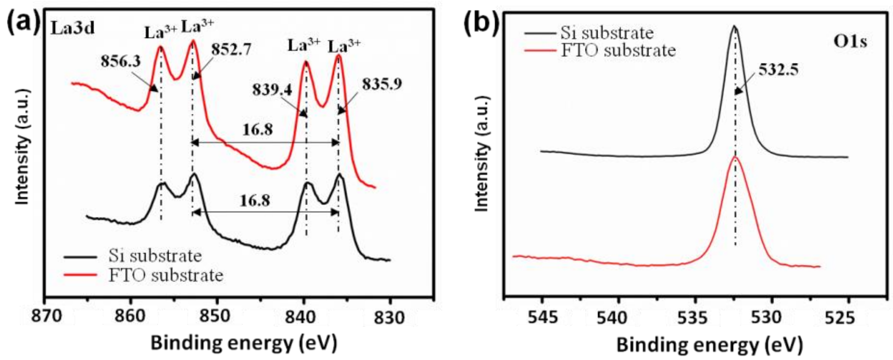

Fig. S8 **a** La 3d and **b** O 1s XPS spectrum of 2.27-nm La_2O_3@NP-NS on FTO and Si substrate

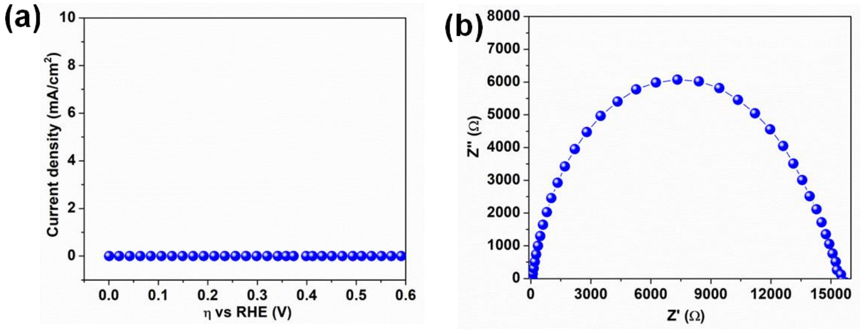

Fig. S9 **a** OER polarization curves of FTO substrate measured in 1 M NaOH aqueous. **b** Nyquist plots of FTO substrate measured in 1 M NaOH solution at a potential of 310 mV vs. RHE

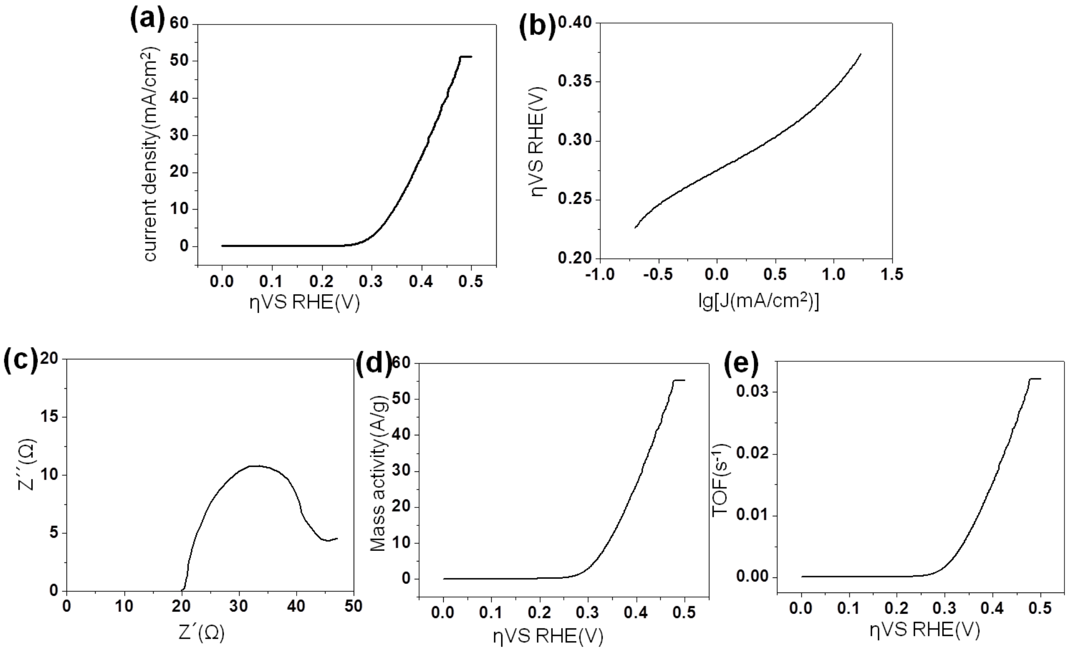

Fig. S10 Electrocatalytic OER performance of commercial IrO_2 powder. **a** OER polarization curves measured in 1 M NaOH solution. **b** Tafel plots. **c** Nyquist plots measured in 1 M NaOH solution at a potential of 310 mV vs. RHE. **d** Mass activity determined from current density as a function of ŋ. **e** TOF determined from j as a function of η

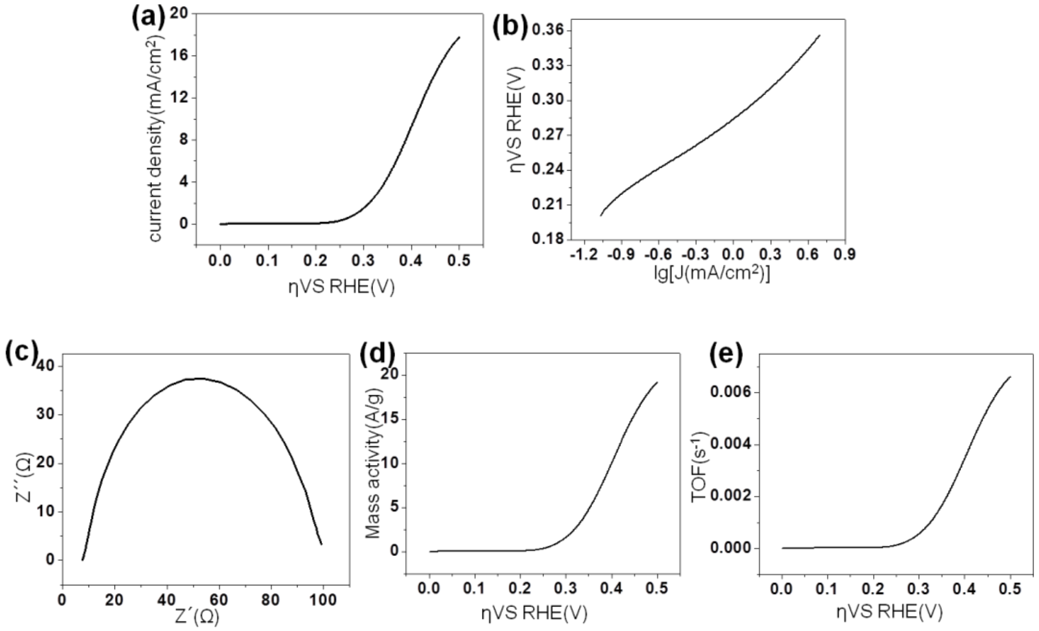

Fig. S11 Electrocatalytic OER performance of commercial RuO_2 powder. **a** OER polarization curves measured in 1 M NaOH solution. **b** Tafel plots. **c** Nyquist plots measured in 1 M NaOH solution at a potential of 310 mV vs. RHE. **d** Mass activity determined from current density as a function of ŋ. **e** TOF determined from j as a function of η

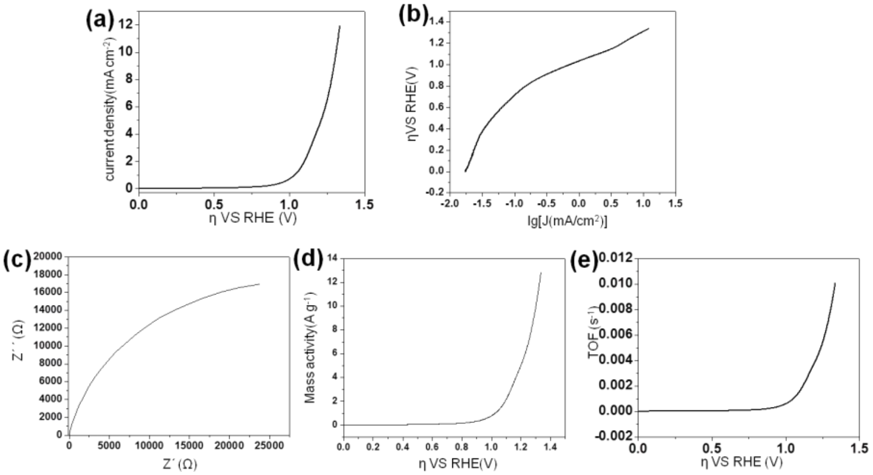

Fig. S12 a OER polarization curves measured in 1 M NaOH solution. **b** Tafel plots. **c** Nyquist plots measured in 1 M NaOH solution at a potential of 310 mV vs. RHE. **d** Mass activity determined from current density as a function of ŋ. **e** TOF determined from j as a function of η

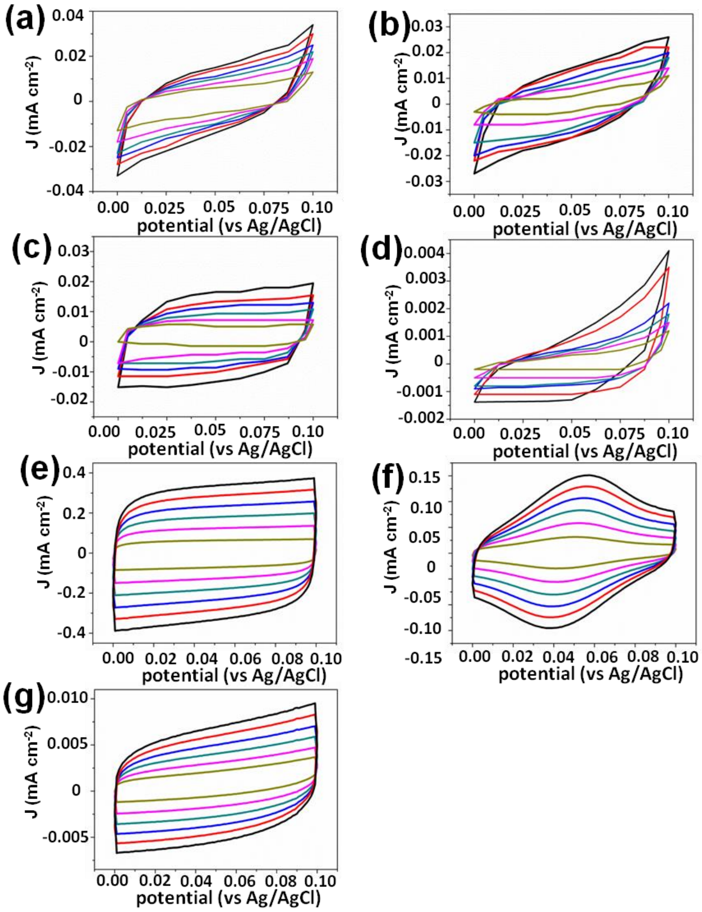

Fig. S13 Cyclic voltammograms curves of of **a** 2.27-nm La_2O_3@NP-NS, **b** 8.68-nm La_2O_3@NP-NS and **c** 28.26 nm thick La_2O_3 nanosheets, **d** ILE synthesized La_2O_3 NPs, commercial **e** IrO_2, **f** RuO_2, and **g** La_2O_3 powder. The scan rates were varied from 10 to 60 mV s^{-1}

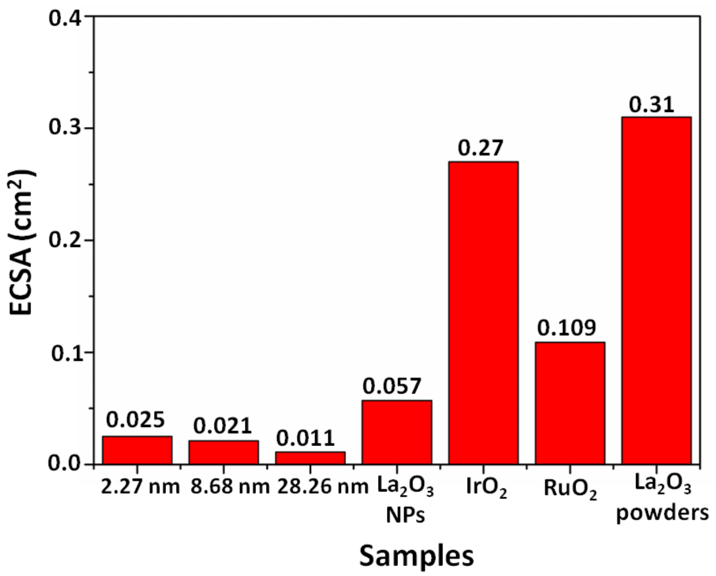

Fig. S14 ECSA of of **a** 2.27-nm La_2O_3@NP-NS, **b** 8.68-nm La_2O_3@NP-NS, and **c** 28.26-nm La_2O_3 nanosheets, **d** ILE synthesized La_2O_3 NPs, commercial **e** IrO_2, **f** RuO_2, and **g** La_2O_3 powder

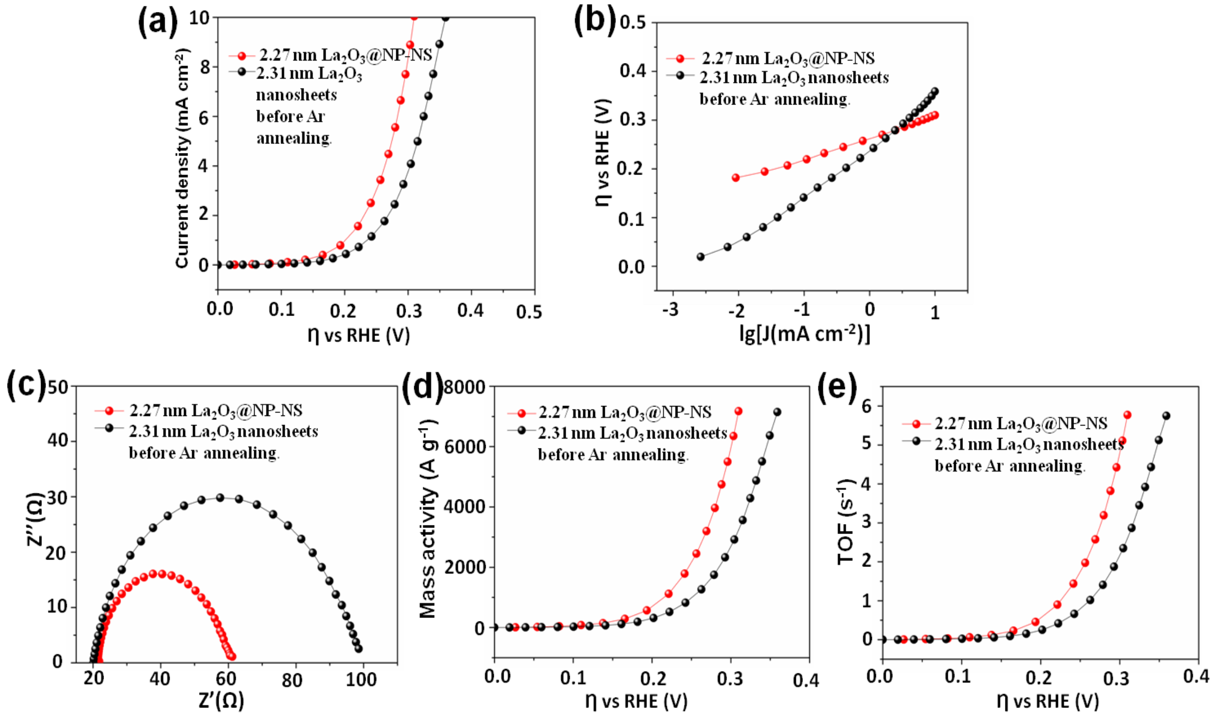

Fig. S15 Electrocatalytic OER performance comparison of 2.27 nm La_2O_3@NP-NS and La_2O_3 nanosheets before Ar annealing. a) OER polarization curves measured in 1 M NaOH solution. b) Tafel plots. c) Nyquist plots measured in 1 M NaOH solution at a potential of 310 mV vs. RHE. d) Mass activity determined from current density as a function of ŋ. e) TOF determined from j as a function of η

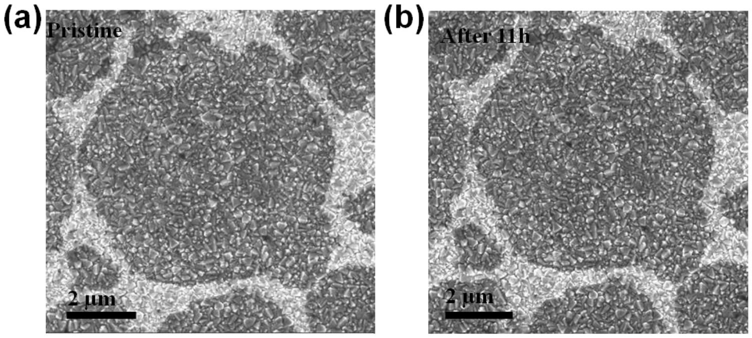

Fig. S16 SEM images of 2.27 nm La_2O_3@NP-NS on FTO substrate before **a** and after **b**11-hour OER

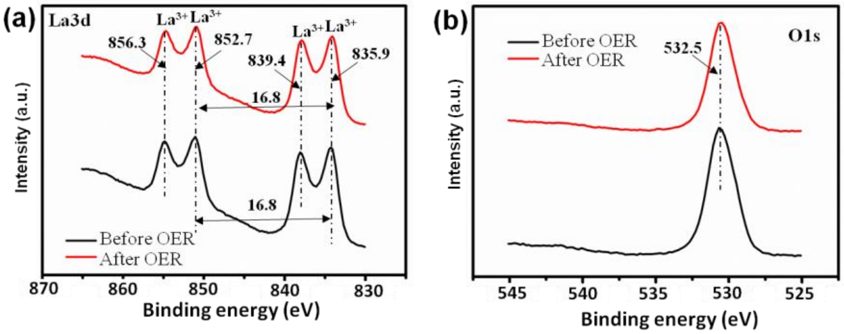

Fig. S17 a La 3d and **b** O 1s XPS spectrum of 2.27 nm La_2O_3@NP-NS on FTO substrate before and after OER measurement

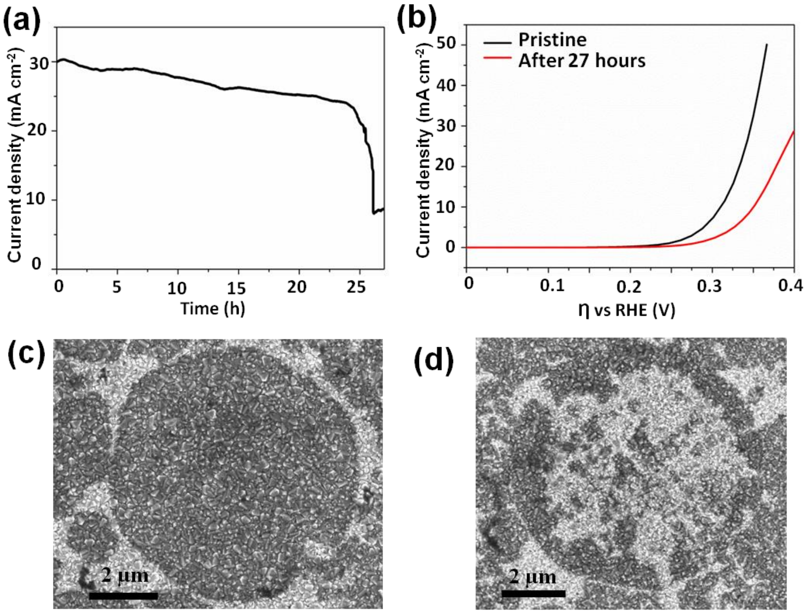

Fig. S18 a Current density measured at η = 345 mV (vs. RHE) as a function of time. **b** OER polarization curves of 2.27-nm La_2O_3@NP-NS before and after 27-hour OER. SEM images of 2.27-nm La_2O_3@NP-NS on FTO substrate before (**c**) and after (**d**) 27-hour OER

Table S1 OER performance comparison between this work and other catalysts

Catalysts	Over potential (mV) @10 mA cm^{-2}	Mass loading (mg cm^{-2})	Mass activity (A g^{-1}) @310 mV	TOF (s^{-1}) @310 mV	Refs.
2.27-nm La_2O_3@NP-NS	310	0.0014	6666.7	5.79	This work
CoFe-LDHs	310	0.2	5.0	-	[S1]
NiFe MOFs	300	-	< 3100	-	[S2]
$Co_2(OH)_3Cl$	270	0.105	286.4	0.73	[S3]
Co_3O_4/CeO_2	270	-	-	< 0.25	[S4]
Co_3O_4	376	-	-	< 0.39	[S5]
NiFeCr	342	-	-	-	[S6]
CoFe LDHs	324	0.02	-	-	[S7]
NiO nanoparticle	335	0.02	72.5	0.0018	[S8]
$Ni_{0.81}Fe_{0.19}O$	310	-	-	<0.26	[S9]
Co_3O_4	339	1.5	3.33	-	[S10]
Ni@NC	390	0.4	2.5	0.136	[S11]
Co_3O_4 NW/CC	320	0.82	9.76	-	[S12]
Ni_2P nanoparticles	290	0.14	142.9	-	[S13]
nNiFe LDH/NGF	337	0.25	-	-	[S14]
IrO_2	338	0.21	12.4	0.022	[S15]
Mn Oxide	540	0.028	71.4	-	[S16]

Supplementary References

[S1] P. Li, M.Y. Wang, X.X. Duan, L.R. Zheng, X.P. Cheng et al., Boosting oxygen evolution of single-atomic ruthenium through electronic coupling with cobalt-iron layered double hydroxides. Nat. Commun. **10**, 1711 (2010). https://doi.org/10.1038/s41467-019-09666-0

[S2] W.R. Cheng, X. Zhao, H. Su, F. Tang, W. Che et al., Lattice-strained metal-organic-framework arrays for bifunctional oxygen electrocatalysis. Nat. Energy **4**, 115-112 (2019). https://doi.org/10.1038/s41560-018-0308-8

[S3] H.L. Jiang, Q. He, X.Y. Li, X.Z. Su, Y.K. Zhang et al., Tracking structural self-reconstruction and identifying true active sites toward cobalt oxychloride precatalyst of oxygen evolution reaction. Adv. Mater. **31**, 1805127 (2019). https://doi.org/10.1002/adma.201805127

[S4] Y. Liu, C. Ma, Q. H. Zhang, W. Wang, P. F. Pan et al., 2D electron gas and oxygen vacancy induced high oxygen evolution performances for advanced Co_3O_4/CeO_2 nanohybrids. Adv. Mater. **31**, 1900062 (2019). https://doi.org/10.1002/adma.201900062

[S5] R. R. Zhang, Y. C. Zhang, L. Pan, G. Q. Shen, N. Mahmood et al., Engineering cobalt defects in cobalt oxide for highly efficient electrocatalytic oxygen evolution. ACS Catal. **8**, 3803-3811 (2018). https://doi.org/10.1021/acscatal.8b01046

[S6] Y. Yang, L. Dang, M.J. Shearer, H.Y. Sheng, W.J. Li, et al., Highly active trimetallic nifecr layered double hydroxide electrocatalysts for oxygen evolution reaction. Adv. Energy Mater. **8**, 1703189 (2018). https://doi.org/10.1002/aenm.201703189

[S7] Y.Y. Wang, C. Xie, Z.Y. Zhang, D.D. Liu, R. Chen et al., In situ exfoliated, n-doped, and edge-rich ultrathin layered double hydroxides nanosheets for oxygen evolution reaction.

Adv. Funct. Mater. **28**, 1703363 (2018). https://doi.org/10.1002/adfm.201703363
[S8] K. Fominykh, G.C. Tok, P. Zeller, H. Hajiyani, T. Miller et al., Rock salt Ni/Co oxides with unusual nanoscale-stabilized composition as water splitting electrocatalysts. Adv. Funct. Mater. **27**, 1605121 (2017). https://doi.org/10.1002/adfm.201605121
[S9] A.C. Pebley, E. Decolvenaere, T.M. Pollocka, M.J. Gordon, Oxygen evolution on fe-doped nio electrocatalysts deposited via microplasma. Nanoscale **9**, 15070 (2017). https://doi.org/10.1039/C7NR04302C
[S10] Y. Zhang, B. Ouyang, J. Xu, G. Jia, S. Chen et al., Rapid synthesis of cobalt nitride nanowires: highly efficient and low-cost catalysts for oxygen evolution. Angew. Chem. Int. Ed. **55**, 8670-8674 (2016). https://doi.org/10.1002/anie.201604372
[S11] J. Ren, M. Antonietti, T. P. Fellinger, Efficient water splitting using a simple Ni/N/C paper electrocatalyst. Adv. Energy Mater. **5**, 1401660 (2015). https://doi.org/10.1002/aenm.201401660
[S12] P. Chen, K. Xu, Z. Fang, Y. Tong, J. Wu et al., Metallic Co_4N porous nanowire arrays activated by surface oxidation as electrocatalysts for the oxygen evolution reaction. Angew. Chem. Int. Ed. **54**, 14710-14714 (2015). https://doi.org/10.1002/anie.201506480
[S13] L.A. Stern, L. Feng, F. Song, X. Hu, Ni_2P as a Janus catalyst for water splitting: the oxygen evolution activity of Ni_2P nanoparticles. Energy Environ. Sci. **8**, 2347-2351 (2015). https://doi.org/10.1039/C5EE01155H
[S14] C. Tang, H.S. Wang, H.F. Wang, Q. Zhang, G.L. Tian et al., Spatially confined hybridization of nanometer-sized NiFe hydroxides into nitrogen-doped graphene frameworks leading to superior oxygen evolution reactivity. Adv. Mater. **27**, 4516-4522 (2015). https://doi.org/10.1002/adma.201501901
[S15] F. Song, X. Hu, Exfoliation of layered double hydroxides for enhanced oxygen evolution catalysis. Nat. Commun. **5**, 4477 (2014).https://doi.org/10.1038/ncomms5477
[S16] Y. Li, P. Hasin, Y. Wu, $NixCo_{3-x}O_4$ Nanowire arrays for electrocatalytic oxygen evolution. Adv. Mater. **22**, 1926-1929 (2010). https://doi.org/10.1002/adma.200903896
